# Self Beyond the Body: Action-Driven and Task-Relevant Purely Distal Cues Modulate Performance and Body Ownership

**DOI:** 10.3389/fnhum.2019.00091

**Published:** 2019-03-20

**Authors:** Klaudia Grechuta, Laura Ulysse, Belén Rubio Ballester, Paul F. M. J. Verschure

**Affiliations:** ^1^Department of Information and Communication Technologies, Universitat Pompeu Fabra, Barcelona, Spain; ^2^Institute for Bioengineering of Catalonia (IBEC), The Barcelona Institute of Science and Technology (BIST), Barcelona, Spain; ^3^Computational Neuroscience Group, Department of Information and Communication Technologies, Center for Brain and Cognition, Pompeu Fabra University, Barcelona, Spain; ^4^Catalan Institution for Research and Advanced Studies (ICREA), Barcelona, Spain

**Keywords:** body ownership, internal forward models, multisensory integration, top-down prediction, goal-oriented behavior, task-relevant cues

## Abstract

Our understanding of body ownership largely relies on the so-called Rubber Hand Illusion (RHI). In this paradigm, synchronous stroking of the real and the rubber hands leads to an illusion of ownership of the rubber hand provided that it is physically, anatomically, and spatially plausible. Self-attribution of an artificial hand also occurs during visuomotor synchrony. In particular, participants experience ownership over a virtual or a rubber hand when the visual feedback of self-initiated movements follows the trajectory of the instantiated motor commands, such as in the Virtual Hand Illusion (VHI) or the moving Rubber Hand Illusion (mRHI). Evidence yields that both when the cues are triggered externally (RHI) and when they result from voluntary actions (VHI and mRHI), the experience of ownership is established through bottom-up integration and top-down prediction of proximodistal cues (visuotactile or visuomotor) within the peripersonal space. It seems, however, that depending on whether the sensory signals are externally (RHI) or self-generated (VHI and mRHI), the top-down expectation signals are qualitatively different. On the one hand, in the RHI the sensory correlations are modulated by top-down influences which constitute empirically induced priors related to the internal (generative) model of the body. On the other hand, in the VHI and mRHI body ownership is actively shaped by processes which allow for continuous comparison between the expected and the actual sensory consequences of the actions. Ample research demonstrates that the differential processing of the predicted and the reafferent information is addressed by the central nervous system via an internal (forward) model or corollary discharge. Indeed, results from the VHI and mRHI suggest that, in action-contexts, the mechanism underlying body ownership could be similar to the forward model. Crucially, forward models integrate across all self-generated sensory signals including not only proximodistal (i.e., visuotactile or visuomotor) but also purely distal sensory cues (i.e., visuoauditory). Thus, if body ownership results from a consistency of a forward model, it will be affected by the (in)congruency of purely distal cues provided that they inform about action-consequences and are relevant to a goal-oriented task. Specifically, they constitute a corrective error signal. Here, we explicitly addressed this question. To test our hypothesis, we devised an embodied virtual reality-based motor task where action outcomes were signaled by distinct auditory cues. By manipulating the cues with respect to their spatial, temporal and semantic congruency, we show that purely distal (visuoauditory) feedback which violates predictions about action outcomes compromises both performance and body ownership. These results demonstrate, for the first time, that body ownership is influenced by not only externally and self-generated cues which pertain to the body within the peripersonal space but also those arising outside of the body. Hence, during goal-oriented tasks body ownership may result from the consistency of forward models.

## 1. Introduction

Humans and other species simultaneously acquire and integrate both self-generated (i.e., reafferent) and externally-generated (i.e., exafferent) information through different sensory channels (Sperry, 1950). Hence, the ability of the nervous system to generate unambiguous interpretations about the body, the so-called body ownership, and determine the source and relevance of a given sensation is fundamental in adaptive goal-oriented behavior (Botvinick and Cohen, 1998; Wolpert and Flanagan, 2001; Van Den Bos and Jeannerod, 2002; Ehrsson, 2012). Imagine playing Air Hockey where the objective is to score points by hitting a puck into the goal. To accomplish the task, at every trial, the brain prepares and generates actions which are most likely to elicit the desired trajectory leading the puck toward the target (Wolpert and Flanagan, 2001; Sober and Sabes, 2003; Shadmehr et al., 2010). Simultaneously, it predicts the sensory consequences of those actions from proprioceptive or tactile modalities which inform about the position and location of the arm, and from visual or auditory modalities which inform about the position and location of the puck (Miall and Wolpert, 1996; Ernst and Bülthoff, 2004; Makin et al., 2008). Since both types of cues may constitute a corrective error signal for the consecutive trial, they are both relevant to the task (Shadmehr et al., 2010; Wolpert et al., 2011). This evidence suggests that the internal models of the external environment, the motor apparatus, and the body are being continuously shaped and updated through sensorimotor interactions of an agent with the world (Miall and Wolpert, 1996; Tsakiris, 2010; Blanke, 2012; Apps and Tsakiris, 2014). Specifically, this tuning occurs through a combination, integration, and prediction of both reafferent and exafferent signals from multisensory sources (Prinz, 1997; Ernst and Bülthoff, 2004; Noë, 2004). However, mechanisms driving the representation of self and, in particular, body ownership in action contexts which require manipulation of the environment and therefore integration of not only proximal or proximodistal but also purely distal cues remain elusive.

In fact, our understanding of body ownership largely relies on the so-called Rubber Hand Illusion (RHI) where subjects passively receive sensory stimuli (Botvinick and Cohen, 1998). RHI is a well-established paradigm (Botvinick and Cohen, 1998; Makin et al., 2008; Tsakiris, 2010) where the illusion of ownership toward a rubber hand emerges during externally-generated synchronous, but not asynchronous, stroking of the real and fake hands (Botvinick and Cohen, 1998). The illusion generalizes to distinct body-parts including fingers, face or a full body (Lenggenhager et al., 2007; Dieguez et al., 2009; Sforza et al., 2010). Initially, Botvinick and Cohen (1998) proposed that the illusion of ownership over the rubber hand is a rather passive sensory state which emerges reactively from a bottom-up integration of multisensory, in this case, visuotactile signals (i.e., proximodistal). Interestingly, subsequent studies investigating mechanisms underlying the RHI extended this classical interpretation by demonstrating that the intermodal matching is not sufficient for the experience of ownership (Tsakiris, 2010). In particular, it has been revealed that the RHI strictly requires physical, anatomical, postural and spatial plausibility of the real and fake hands (Tsakiris and Haggard, 2005; Costantini and Haggard, 2007; Lloyd, 2007; Makin et al., 2008) (see also Liepelt et al., 2017). Hence, the bottom-up integration of multisensory inputs seems to be modulated by experience-driven predictive information, which allows for active comparison between the properties of the viewed (non)-corporeal object and the internal model of the body (Tsakiris et al., 2008; Apps and Tsakiris, 2014). The finding of Ferri et al. (2013, 2017) further supported the fundamental role of the top-down processes in the modulation of body ownership. The authors demonstrated that the experience of ownership over a non-bodily object could originate as a consequence of pure expectation and anticipation of correlated exafference in the absence of actual tactile stimulation (Ferri et al., 2013, 2017). Together, this evidence supports the hypothesis that in the context of externally generated inputs (classical RHI), body ownership relies on two intertwined processes. Namely, (1) the bottom-up accumulation and integration of tactile and visual cues, and (2) top-down comparison between the novel sensory stimuli (i.e., rubber hand) and experience-driven priors about the internal model of the body (Tsakiris and Haggard, 2005; Blanke, 2012; Clark, 2013; Seth, 2013; Apps and Tsakiris, 2014). We will refer to tactile or proprioceptive modalities as *proximal*, requiring an object to enter in direct contact with the surface of the body, and to the visual or auditory modalities as *distal*, sensing from a distance without getting in direct contact with the body.

Only recently the principles of body ownership have been studied in the context of self-generated (reafferent) sensory signals using physical set-ups (i.e., moving Rubber Hand Illusion, mRHI) (Tsakiris et al., 2006; Dummer et al., 2009; Kammers et al., 2009; Newport et al., 2010; Walsh et al., 2011; Ma and Hommel, 2015a,b), or virtual reality (i.e., moving Virtual Hand Illusion, VHI) (Sanchez-Vives et al., 2010; Kalckert and Ehrsson, 2012; Shibuya et al., 2018). In these protocols which include movement (mRHI and VHI), subjects are typically instructed to reach a specific target (goal-oriented) or to move the fingers/hand/arm continuously within a specific area (free exploration) while observing the (a)synchronously moving rubber or virtual analog. The results yield that there is a strong experience of ownership in the condition where the movements of the real and fake arms are spatiotemporally aligned (Dummer et al., 2009). Contrarily, participants report no ownership of the fake body-part when the visual feedback of self-initiated movement is (inconsistently) delayed or displaced, and therefore does match the proprioceptive information (Blakemore et al., 2000). Hence, similar to the classical RHI, in the context of self-generated movements, ownership seems to depend on the consistency of sensory information from proximodistal modalities, in this case, proprioceptive (proximal) and visual (distal). Interestingly, different to the classical RHI, in VHI as well as mRHI the experience of ownership emerges independently of whether (1) the visual, anatomical or structural properties of the avatar satisfy well-established priors about the own body (Banakou et al., 2013; Peck et al., 2013; Ma and Hommel, 2015a; Romano et al., 2015; Van Dam and Stephens, 2018), (2) there is a (consistent) delay in the visual feedback of the movement (3) the viewed object is ‘connected' to participants' body (Ma and Hommel, 2015a). Crucially, the condition which needs to be satisfied is that the action-driven sensory feedback from proximodistal modalities matches the predicted one (Dummer et al., 2009; Sanchez-Vives et al., 2010; Ma and Hommel, 2015b). In line with physiological and motor control studies (Miall and Wolpert, 1996; Wolpert and Flanagan, 2001; Proske and Gandevia, 2012), this evidence suggests that when moving in a goal-oriented manner body ownership is weighted stronger by the congruency of the internal (forward) model of the action and the action effects, the same mechanism which impacts agency (Gallagher, 2007; Hommel, 2009; Longo and Haggard, 2009; D'Angelo et al., 2018), rather than the (generative) model of the body and its physical specifics Ma and Hommel (2015b). Crucially, it has been well established that the forward models are not limited to the bodily (proximal or proximodistal) feedback exclusively, but instead, they integrate across all sensory predictions which pertain to the interactions of an agent within an environment, including purely distal cues (Jordan and Rumelhart, 1992; Miall and Wolpert, 1996). For instance, under normal conditions, the visuoauditory signals of the puck hitting the goal are spatiotemporally aligned with its trajectory that depends on the direction of the arm movement. However, if the actual location of the sound of the puck hitting the goal does not correspond to the efference copy or corollary discharge, it would reflect on the Sensory Prediction Errors (SPE) of the forward model (Wolpert et al., 1995, 2011; Miall and Wolpert, 1996; Woodgate et al., 2015; Maffei et al., 2017). Thus, if body ownership results from a consistency of forward models, it would be affected by the (in)congruency of not only proximodistal cues such as in the mRHI and VHI (Dummer et al., 2009; Sanchez-Vives et al., 2010) but also purely distal signals given that they constitute task-relevant information about the action-consequences.

Here, we propose that in contexts where the sensory signals are self-generated, such as in the moving Rubber Hand Illusion or the Virtual Hand Illusion, body ownership depends on the sensory prediction errors from purely distal multisensory modalities, which would suggest a mechanism similar to the forward model or corollary discharge. We, therefore, hypothesize that the experience of ownership over a virtual body will be compromised when action-driven and task-relevant visuoauditory feedback of goal-oriented movements will not match sensory predictions. We also expect that the incongruency of those cues will affect performance. To test this hypothesis, we devise an embodied virtual reality-based goal-oriented task where action outcomes are signaled by distinct auditory signals. We manipulate the cues with respect to their spatial, temporal and semantic congruency, and compare body ownership and performance across two experimental conditions, where purely distal cues are either congruent or incongruent. Our results demonstrate, for the first time, that purely distal signals which violate predictions about the consequences of action-driven outcomes affect both performance and body ownership.

## 2. Materials and Methods

### 2.1. Participants

After providing written informed consent, sixteen healthy participants were recruited for the study, eight males (mean age 24.0±2.65) and eight females (mean age 22.64±2.25). Since no previous study assessed the effects of the congruency of purely distal modalities on body ownership, we could not perform a power analysis to determine the sample size. We, therefore, based the choice of *N* on previous studies (Mohler et al., 2010). All subjects were right-handed (handedness assessed using the Edinburgh Handedness Inventory) (Oldfield, 1971), had normal or corrected-to-normal vision and reported normal hearing. They were pseudorandomly assigned to two experimental groups following a between-subjects design, which prevented habituation to the ownership measures, visuoauditory manipulations, and fatigue. We used stratified randomization to balance the conditions in terms of age, gender and previous experience with virtual reality. All participants were blind to the purpose of the study. The experimental procedures were previously approved by the ethical committee of the University of Pompeu Fabra (Barcelona, Spain).

### 2.2. Task: Virtual Reality-Based Air Hockey.

**Figure 1:**
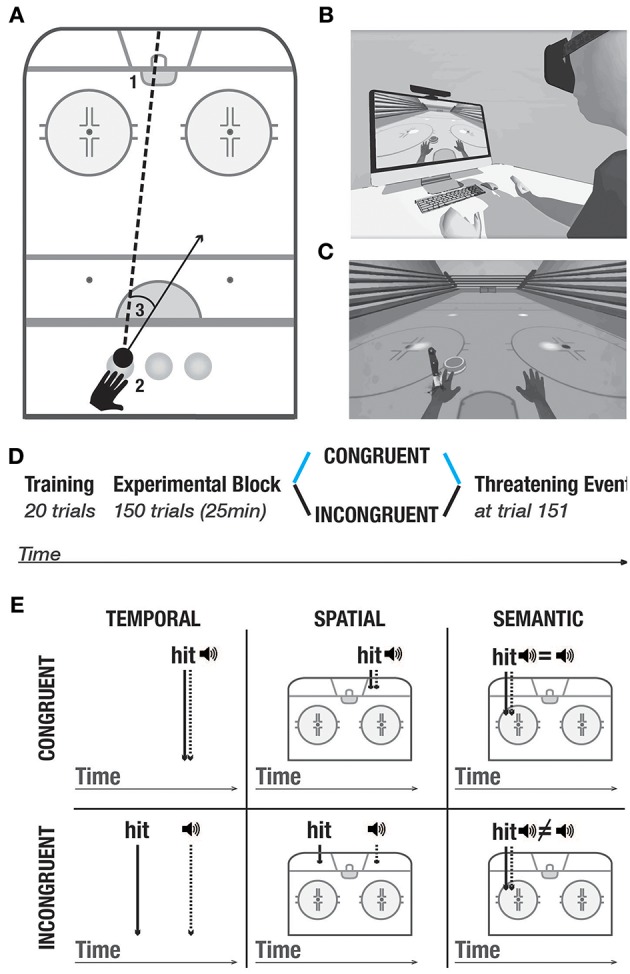
Experimental setup and protocol. **(A)** Task. 1- goal. 2- three starting positions. 3- example of a directional error, calculated as the difference between the actual direction vector and a straight line between the position of the puck and the goal. **(B)** Experimental setup. **(C)** Threatening event. **(D)** Experimental protocol. All participants underwent the training block. In the experimental block, they were randomly split into two conditions: Congruent (blue), and incongruent (black). At trial 151, all participants went through the threatening event which served to measure galvanic skin responses. The same color-code (congruent- blue, incongruent- black) is used in the following figures. **(E)** Purely distal visuoauditory manipulations- temporal, spatial and semantic. Upper panel: congruent condition; lower panel: incongruent condition.

The experimental setup (Figure 1A) comprised a personal computer, a motion detection system (Kinect, Microsoft, Seattle), a Head Mounted Display (HTC Vive, www.vive.com) and headphones.

Similar to others (Sanchez-Vives et al., 2010; Grechuta et al., 2017), here we used virtual reality as a tool to study the modulation of body ownership. The protocol was integrated within the virtual environment of the Rehabilitation Gaming System (Cameirão et al., 2010; Grechuta et al., 2014). During the experiment, while seated at a table, participants were required to complete a goal-oriented task that consisted in hitting a virtual puck into the goal (air hockey, Figure 1A, B1). The virtual body was spatially aligned to the real body. Throughout the experiment, the participants' arm movements were continuously tracked and mapped onto the avatar's arms, such that the subjects interacted with the virtual environment by making planar, horizontal movements over a tabletop (Figures 1A, B). To prevent repetitive gestures, at the beginning of every trial the puck pseudorandomly appeared in one of the three starting positions (left, center, right) (Figure 1 B2). The frequency of appearance of every starting position was uniformly distributed within every experimental session. Participants received instructions to place their hand in an indicated starting position and to execute the movement to hit the puck when its color changed to green (“go” signal). Each trial consisted of one “hit” which could end in either a success (the puck enters the goal) or a failure (the puck hits one of the three walls). At the end of every trial, participants were to place their left hand back at the starting position. The experimental block, in both conditions, consisted of 150 trials preceded by 20 trials of training (training block) (Figure 1D) and followed by a threatening event. The threatening event served to measure autonomous responses to an unexpected threat (body ownership measure, Figure 1C) (Armel and Ramachandran, 2003). Overall, the task had an approximate duration of 20 min.

### 2.3. Multisensory Feedback.

*Task-Relevant Visuomotor Signals*. Throughout the experiment, participants were exposed to the visual feedback of self-generated arm movements. Specifically, the real arms were tracked by the motion detection input device and mapped onto the avatar's arms in real time allowing synchronous feedback. This method served to control for the congruency of proximodistal (i.e., visuomotor) signals which has been shown to underlie body ownership and agency (Sanchez-Vives et al., 2010). It also guaranteed that the only manipulated variables were the distal modalities (i.e., visual and auditory).

*Task-Relevant Visuoauditory Signals*. The task included task-relevant distal cues in the form of auditory feedback which was triggered as a consequence of every interaction of the puck with the environment. In particular, at the end of every trial, an auditory cue constituted a binary reinforcement signal informing about a failure (negative sound) or a success (positive sound). To study whether purely distal cues influence body ownership and performance, we manipulated the congruency of the auditory stimuli in three domains (Figure 1E) — temporal: the time of the cue was synchronized with the time of the hit; spatial: the cue originated from the location of the hit, and semantic: the feedback of the cue reflected performance in a binary way (i.e., success or failure). The auditory cues were manipulated in two experimental conditions including congruent and incongruent. In the training block and the congruent condition, auditory cues were always congruent such that they occurred at the time of the hit, at the location of the hit, and they reflected performance. In the incongruent condition, the auditory signals were always incongruent. Namely, (1) the sound of the hit was anticipated or delayed, that is, it occurred randomly within 200–500 ms before or after the actual collision (temporal domain), (2) it originated in a different location than the actual hit, that is, 5–15° away from the actual hit, or (3) it did not reflect performance, that is participants heard the sound of failure following a successful trial and vice versa (semantic domain).

We chose those three manipulations to include all the dimensions necessary for the performance of the present task: direction, force, as well as the knowledge of results. Each of the dimensions (spatial, temporal, and semantic) provides unique information to the subject about the consequences of one's actions. Specifically, (1) the spatial dimension informs about the direction of the ballistic movement (where the puck hits the wall/goal), (2) the temporal dimension informs about the force applied to the action (when the puck reaches the wall/goal), whereas (3) the semantic dimension informs about the outcome of the action (either success or a failure). As such, all these dimensions contribute to the generation of prediction errors that can be integrated by an internal model to adjust motor performance. Spatial and temporal dimensions provide information about the action parameters on a continuous range and can be used as a supervising signal whereas the semantic dimension constitutes a binary reinforcement signal. All manipulations were pseudorandomly distributed and counterbalanced within each session to counteract order effects. Importantly, task-relevant proximodistal cues such as the visual feedback of the arm movements remained congruent in both conditions.

### 2.4. Measures

#### 2.4.1. Motor Control

We used three measures to quantify performance: scores, directional error, and reaction times. Scores were calculated as the percentage of successful trials (the puck enters the gate), while the directional error indicated the absolute angular deviation from the straight line between the starting position of the puck (left, central or right) and the center of the gate (Figure 1, B3). We computed the reaction times as time intervals between the appearance of the puck and action initiation. Since the task did not impose a time limit, we expected neither significant differences in reaction times between the conditions nor speed-accuracy trade-offs. We predicted that the manipulations of purely distal (visuoauditory) action-driven signals in the incongruent condition might alter scores and directional accuracy as compared to the congruent condition.

#### 2.4.2. Body Ownership

*Galvanic Skin Response (GSR)*. At the end of every experimental session, in both conditions, we introduced a threatening event (a knife falling to stab the palm of the virtual hand, Figure 1C) to quantify autonomous, physiological responses to an unexpected threat (Armel and Ramachandran, 2003). To prevent movement-driven muscular artifacts, we recorded the skin conductance responses from the right hand which did not move during the experiment. For the analysis, we calculated the mean and the standard deviation of the integral of the baseline (10 s time window before the threatening stimulus onset)-subtracted signal per condition in a non-overlapping time windows of 9 s (Petkova and Ehrsson, 2008). In particular, we expected an increase in the GSRs following the threatening stimulus in the congruent as compared to the incongruent condition.

*Proprioceptive drift*. Prior to and upon completion of the experiment, all the subjects completed the proprioceptive drift test which followed a standard technique, see for instance (Sanchez-Vives et al., 2010). Specifically, the participants were asked to point to the location of the tip of their left index finger with the right index finger with no visual feedback available. The error in pointing (Tsakiris and Haggard, 2005) was computed as the distance between the two locations (the actual location of the tip of the left index finger and the pointing location) and measured in centimeters. We subtracted baseline responses from post-experimental errors for each participant. We expected stronger proprioceptive recalibration, and therefore, higher pointing errors in the congruent as compared to the incongruent condition.

*Self-reports*. At the end of every session, all participants completed a questionnaire which evaluated the subjective perception of body ownership and agency, adapted from a previous study (Kalckert and Ehrsson, 2012). The entire questionnaire consisted of twelve items (Table 1), six per domain (ownership and agency), three of which were related to the experience of ownership and agency, respectively, while the remaining served as controls. Participants answered each statement on a 7-point Likert Scale ranging from “–3”: being in strong disagreement to “3”: being in strong agreement. To counteract order effects, the sequence of the questions was randomized across all the subjects.

**Table 1:** The questionnaire, consisting of 12 statements divided into four different categories.

**Category**	**Question**
Ownership	I felt as if I was looking at my own hand
	I felt as if the virtual hand was part of my body
	I felt the virtual hand was my hand
Ownership control	It seemed as if I had more than one left hand
	It appeared as if the virtual hand were drifting toward my real hand
	It felt as if I had no longer a left hand, as if my left hand had disappeared
Agency	The virtual hand moved just like I wanted it to, as if it was obeying my will
	I felt as if I was controlling the movements of the virtual hand
	I felt as if I was causing the movement I saw, and the control questions were
Agency control	I felt as if the virtual hand was controlling my will
	I felt as if the virtual hand was controlling my movements
	I could sense the movement from somewhere between my real and virtual hand

## 3. Results

To test our hypothesis that action-driven purely distal cues which pertain to the task contribute to body ownership, we used a virtual reality-based experimental setup (Figures 1A, B) where subjects were to complete a goal-oriented task, and manipulated the congruency of auditory action outcomes (Figure 1E). The experimental protocol (Figure 1D) consisted of three phases: the training block, (2) the experimental block in either congruent or incongruent condition, and (3) the threatening event (Figures 1D, C). To quantify body ownership, for each experimental session, we measured proprioceptive drifts, recorded Galvanic Skin Responses (GSR) to an unexpected threat, and administered self-reports. To measure performance, we computed scores, directional errors, and reaction times. For the analysis, we used *t*-tests and calculated Cohen's d to evaluate differences between conditions and the associated effect sizes.

### 3.1. Motor Control

**Figure 2:**
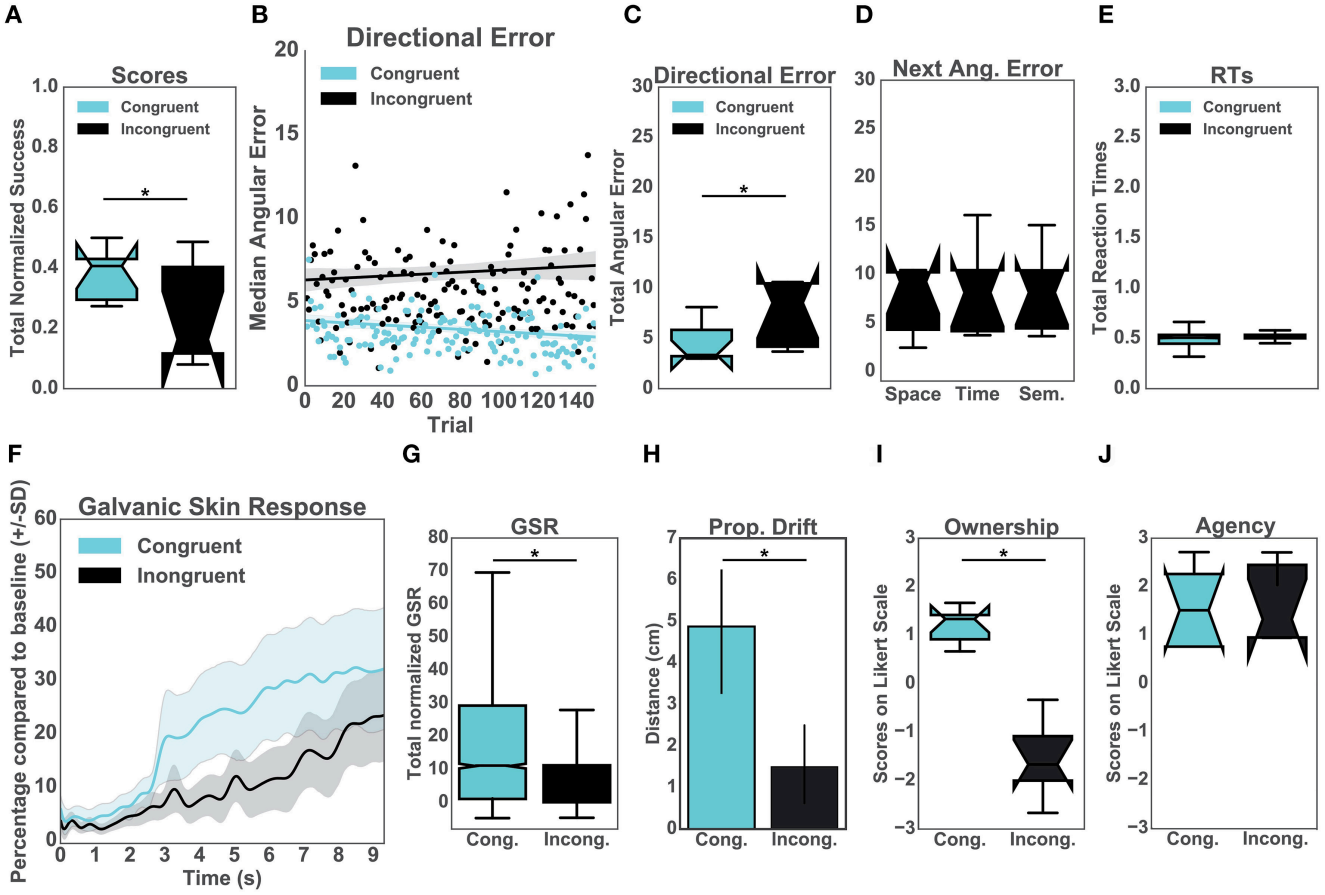
**Upper panel**: Performance. **(A)** Normalized percentage of successful trials per group. **(B)** Median directional error per trial over the experimental block (*N* = 150) split per condition **(C)** Total directional error from all the trials per subject per condition. **(D)** This graph represents the mean values for the incongruent group only. In particular, the effects of the three auditory manipulations (spatial, temporal, and semantic) on the mean directional error on the consecutive trials. **(E)** Mean reaction times from all trials per condition. **Lower panel**: Body Ownership. **(F)** Galvanic Skin Response (GSR). The sampling rate for the GSR signal was 60 Hz. Accordingly, the data was run through a low-pass filter with a cut-off frequency of 3 Hz. The plot represents the mean GSR and the associated standard deviation for all participants in a time window of 9 s (Hägni et al., 2008), split per condition. The threatening event happened at time 0. **(G)** Mean GSR from 9 s post threatening event. **(H)** Proprioceptive drift. Results of the difference between pre- and post-test calculated in centimeters per condition. **(I)** Score from the self-reported experience of body ownership per group. Scores above 0 indicate ownership. **(J)** Score from the self-reported experience of agency per group. Scores above 0 indicate the experience of agency.

Firstly, our results showed that the normalized performance-scores (proportion of successful trials) were significantly higher in the congruent (μ = 0.35, *sd* = 0.47) than in the incongruent condition (μ = 0.17, *sd* = 0.38), [*t*_(14)_= 8.89, *p* < 0.001, *d* = 0.42] (Figure 2A). To explore the effects of the congruency of purely distal signals on performance, we compared both conditions in terms of directional errors (Figure 2B). In particular, a T-test indicated that the errors were significantly higher in the incongruent (μ = 6.42, *sd* = 4.52) than in the congruent condition (μ = 3.30, *sd* = 2.01), [*t*_(14)_ = 19.52, *p* < 0.001, *d* = 0.89] (Figure 2C). To further investigate the relationship between the quality of the distal cues and performance, we averaged and compared the directional errors following the three types of auditory manipulations (Figure 2D). This analysis was performed exclusively for the incongruent condition. Interestingly, we found no difference between the distinct auditory cues including spatial (μ = 10.17, *sd* = 13.33), temporal (μ = 7.99, *sd* = 9.75) and semantic (μ = 7.22, *sd* = 7.23) cues (Figure 2D). Specifically, a Kruskal-Wallis test indicated that all manipulations had the same significant effect on body ownership [*x*^2^_(2)_ = 1.74, *p* = 0.39]. In addition, we observed that the congruency of the distal cues had no significant effect on the averaged reaction times when comparing the incongruent group (μ = 0.48, *sd* = 0.05) with the congruent group (μ = 0.51, *sd* = 0.01), *p* = 0.46 (Figure 2E).

### 3.2. Body Ownership

Prior to the appearance of the knife (10s baseline), the skin conductance was not different between the two groups [*t*_(14)_ = 0.60, *p* = 0.55; μ = 181.12, *sd* = 112.43 for the congruent condition and μ = 230.25, *sd* = 183.75 for the incongruent condition]. The analysis revealed, however, that the post-threatening stimulus GSR was significantly higher in the congruent (μ = 42.54, *sd* = 33.98) than in the incongruent group (μ = 29.67, *sd* = 26.82) [*t*_(14)_ = 21.03, *p* < 0.001, *d* = 0.42] (Figures 2F, G). Similarly, we found a difference in the proprioceptive drift between the congruent (μ = 4.88, *sd* = 2.36) and incongruent group (μ = 1.5, *sd* = 1.51) such that the errors in were significantly higher in the congruent condition [*t*_(14)_ = 3.4, *p* = 0.004, *d* = 1.7] (Figure 2H). We further report a statistically significant difference in the self-reported experience of ownership between the two conditions [*t*_(14)_ = 4.97, *p* < 0.001, *d* = 2.5]. The ownership ratings in the congruent group (μ = 1.13, *sd* = 0.56) were greater than in the incongruent group (μ = −1.3, *sd* = 1.25). We found no difference between the congruent (μ = −1.33, *sd* = 1.46) and the incongruent group (μ = −1.3, *sd* = 1.25) for the three control items [*t*_(14)_ = 1.79, *p* = 1.38]. We later analyzed questions related to agency. The results showed differences neither for the control questions [*t*_(14)_ = 0.22, *p* = 0.82] between congruent (μ = −1.67, *sd* = 1.49) and incongruent condition (μ = −1.83, *sd* = 1.48) nor for the experimental ones, congruent (μ = 1.5, *sd* = 1.13) and incongruent condition (μ = 1.33, *sd* = 1.48). In both groups participants experienced high agency during the experiment.

### 3.3. Relationship of the Ownership Measures

**Figure 3:**
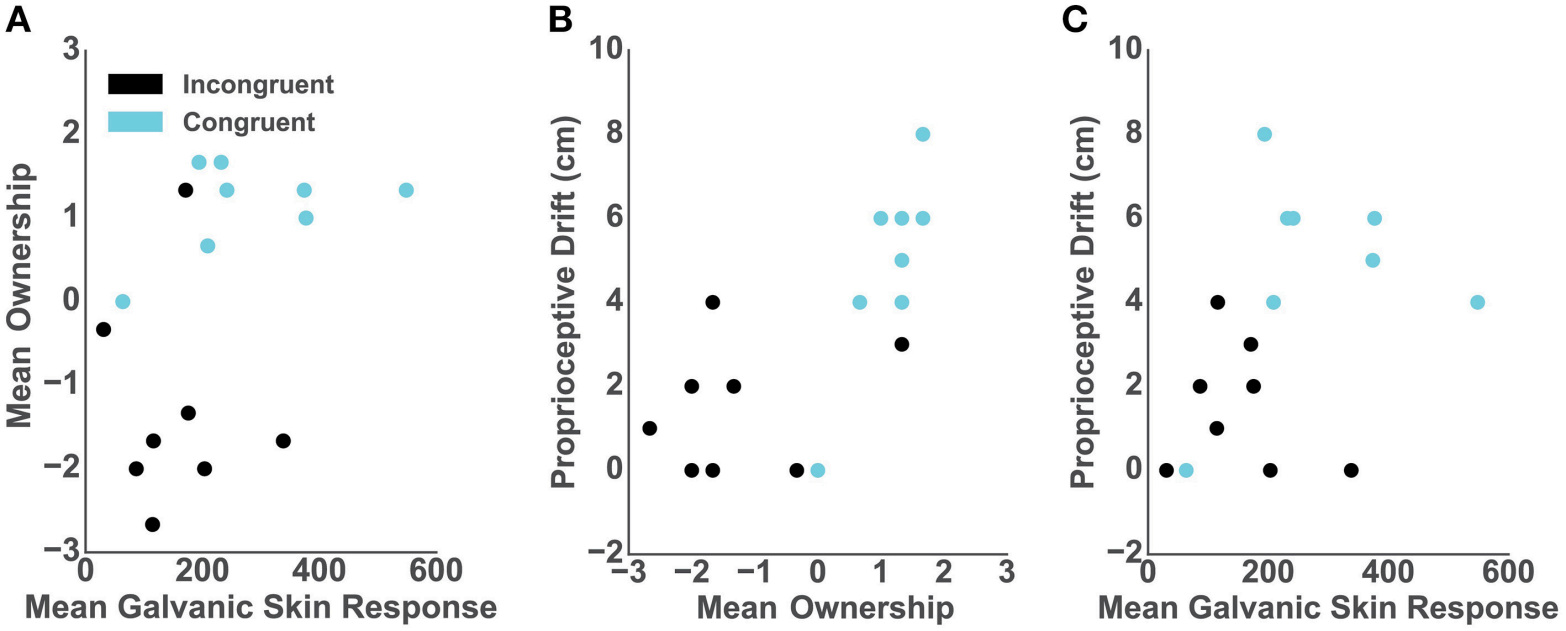
Correlations. In all graphs dots represent individual participants and colors represent conditions: blue- congruent and black- incongruent. **(A)** Mean GSR 9 s post-threatening event and mean self-reported ownership. **(B)** Mean self-reported ownership and the proprioceptive drift score. **(C)** Mean GSR 9 s post-threatening event and the proprioceptive drift score.

We assessed the relationship between the objective, subjective and behavioral ownership measures and, per each participant in both conditions, we computed: (1) mean GSR from 9 s post-threat, (2) mean of the three ownership questions; and (3) baseline-subtracted proprioceptive drift. The Spearman rank-order correlation between post-threat GSR and self-reported ownership was close to significance (*r* = 0.47; *p* = 0.06) (Figure 3A). However, we report high and significant positive correlation between the proprioceptive drift and self-reported ownership (*r* = 0.75; *p* < 0.001) (Figure 3B) as well as between the post-threatening GSR and proprioceptive drift (*r* = 0.52; *p* < 0.03) (Figure 3C).

## 4. Discussion

In this study, we asked whether body ownership depends on the consistency of task-relevant purely distal sensory cues which result from self-initiated actions. In particular, we investigated the influence of those cues on performance and ownership using an embodied, virtual reality-based goal-oriented task where action outcomes were signaled by distinct auditory signals. We manipulated the congruency and therefore the predictability of those reafferent sensory signals and hypothesized that the (in)congruency of visuoauditory stimuli would affect both performance and body ownership. Our results support our prediction and suggest that both are compromised when action-driven purely distal signals are incongruent.

The plasticity of body ownership relative to the spatiotemporal coincidence of exafferent and reafferent multisensory signals has been well-accepted (Botvinick and Cohen, 1998; Craig, 2002; Tsakiris, 2010; Blanke, 2012; Seth, 2013; Suzuki et al., 2013). In particular, neurophysiological and behavioral studies have demonstrated that the experience of ownership is established through bottom-up integration and top-down prediction of proximodistal cues within the peripersonal space (Rizzolatti et al., 1981; Makin et al., 2007; Tsakiris, 2010; Blanke, 2012). Crucially, however, depending on whether the sensory signals are externally (classical Rubber Hand Illusion, RHI) or self-generated (*moving* Rubber Hand Illusion, mRHI and moving Virtual Hand Illusion, VHI), the top-down expectation signals seem to be qualitatively different. On the one hand, in the RHI, the sensory correlations are modulated by top-down influences which constitute empirically induced priors related to the internal model of the body (Tsakiris and Haggard, 2005; Costantini and Haggard, 2007; Lloyd, 2007; Makin et al., 2008). For instance, the illusion of ownership will not occur if the shape or the location of the fake hand is not plausible (Tsakiris, 2010). On the other hand, the evidence from the mRHI and VHI supports that, in the contexts of self-generated stimuli, body ownership is actively shaped by top-down processes allowing for continuous comparison between the actual and predicted action-consequences from proximodistal modalities (Dummer et al., 2009; Sanchez-Vives et al., 2010; Ma and Hommel, 2015a,b). In fact, when the errors in those sensory predictions (the so-called Sensory Prediction Errors, SPE) are insignificant, that is, when the visual feedback of the position of the rubber (mRHI) or virtual (VHI) hand is congruent with the proprioceptive cues, the ownership over the artificial arm is high, and vice versa (Dummer et al., 2009; Sanchez-Vives et al., 2010). Contrary to the standard RHI, in the mRHI and VHI, the physical, spatial and temporal characteristics of the body do not influence the experience of ownership (Banakou et al., 2013; Peck et al., 2013; Ma and Hommel, 2015b; Romano et al., 2015; Van Dam and Stephens, 2018). Moreover, it has been demonstrated that participants can perceive an actively operated virtual non-corporeal and ‘disconnected' object (balloon or a square) as an extension of their own body as long as it follows the predicted trajectory Ma and Hommel (2015a). Thus, when acting in the world, the top-down predictive processing modulating ownership seems not to depend on the generative models of self but rather on the forward models (or corollary discharge) which guide action by generating sensory predictions about the consequences of movement based on the efference copy (Miall and Wolpert, 1996; Sanchez-Vives et al., 2010; Ma and Hommel, 2015b; Kilteni and Ehrsson, 2017). Similar, from the perspective of ideomotor theory, ownership might be viewed as depending on the difference between the goals (intended action effects) and the perceptual consequences (actual action effects) (Stock and Stock, 2004; Hommel, 2009; Shin et al., 2010).

Ample research demonstrates that the central nervous system uses forward models for the differential processing of the predicted and the actual reafferent information which was shown to underlie motor control and agency (Wolpert et al., 1995; Miall and Wolpert, 1996; Bäß et al., 2008; Crapse and Sommer, 2008; Sommer and Wurtz, 2008; Schwarz et al., 2018). Crucially, the internal (forward) models do not exclusively process sensory signals related to the body, but they integrate across all sensory information from both proximal (proprioceptive, tactile) and distal (visual, auditory) modalities (Jordan and Rumelhart, 1992; Miall and Wolpert, 1996). This would suggest that, if body ownership results from a consistency of forward models, it will be affected by the(in)congruency of not only proximodistal cues such as in the moving rubber hand illusion or the virtual hand illusion (Dummer et al., 2009; Sanchez-Vives et al., 2010) but also purely distal signals given that they constitute information about the action-consequences. In this study, we explicitly addressed this question using a variation of a VHI paradigm, which required the participants to perform actions that triggered distal (auditory) cues. Those auditory cues indicated the location and the time of a collision of a puck with the walls or the goal as well as the outcome (failure or success). To test whether action-driven and task-relevant sensory signals impact body ownership, in one of the groups, we manipulated their congruency. We predicted that the ownership scores, measured subjectively, objectively and behaviorally could be lower in the condition where the cues do not match predictions about purely distal sensory signals.

Did the proposed purely distal cues affect body ownership? Results from all the ownership measures (Figure 2, Lower panel: Body Ownership), including skin conductance (GSR), proprioceptive drift and the questionnaire support that purely distal cues which pertain to the task and violate predictions about the auditory action outcomes compromise body ownership. Specifically, we found that the scores were significantly higher in the congruent compared to the incongruent condition in all analyses (Figure 2, Lower panel: Body Ownership). Subsequent correlations between the proposed measures (Figure 3) further confirmed the consistency of the obtained results within three dimensions of ownership quantification including physiological response, behavioral proprioceptive recalibration, and a conscious report (Longo et al., 2008). Similar to the mRHI, VHI (Sanchez-Vives et al., 2010; Ma and Hommel, 2015b) and their variations (i.e., Ma and Hommel, 2015a), here we interpret the obtained low-ownership outcome in the incongruent condition (Figure 2, Upper panel: body Ownership) as a consequence of high sensory prediction errors possibly computed but the forward model (Miall and Wolpert, 1996; Crapse and Sommer, 2008; Limanowski and Blankenburg, 2013; Apps and Tsakiris, 2014). In our case, however, the sensory conflicts were driven by a discrepancy between the predicted and actual purely distal visuoauditory signals which did not pertain to the body but were relevant to the outcome of the goal-oriented task. We speculate that the manipulation of the proposed signals might have reflected on the errors of the forward models which influence performance and possibly body ownership (Wolpert et al., 1995). This could further suggest that the integration of signals from distal modalities might affect the integration of signals from proximal or proximodistal modalities establishing a feedback loop. In such case, any (in)congruent relationship between distal, proximodistal, and proximal signals which pertain to the goal of the task would affect the experience of ownership and even define the boundaries of the embodied self. To the best of our knowledge, our results propose for the first time that the ownership of a body might be driven by bottom-up integration and top-down prediction of purely distal modalities occurring outside of the body and outside of the peripersonal space (Rizzolatti et al., 1981). This would support recent findings which suggest that body ownership is coupled to the motor systems and that, similar to the experience of agency, it might depend on the congruency of a forward model or corollary discharge (Ma and Hommel, 2015b; Grechuta et al., 2017; Kilteni and Ehrsson, 2017). As expected, the visuoauditory manipulations did not significantly influence the perceived agency (Figure 2J). Participants reported control over the virtual hand in both conditions, probably due to the congruent mapping of the proximal cues (see Methods section about the sensory manipulations). The visual feedback of the movement of the arm always followed the desired trajectory, which is one of the three questions addressed in the standard self-reported agency assessment (Kalckert and Ehrsson, 2012).

At the current stage, two questions remain open. First, how can the integration of distal and proximodistal cues occur in the service of body ownership? Since the primary purpose of the present study was to investigate the influence of purely distal signals on body ownership, the proximodistal (visuo-proprioceptive) cues within the peripersonal space were congruent in both groups. Indeed, based on those cues, participants could always predict the location and the time of the distal auditory signals (spatial and temporal manipulation) as well as the outcome of an action (semantic manipulation). Therefore, in the incongruent condition, where the distal consequences of the actions did not match the predictions, we expected that the sensory prediction errors would negatively impact ownership. However, with the current design, we can neither explain the interaction of the proximodistal and distal cues nor how do they weight the experience of ownership. Future studies should further investigate the relationship between the visual and auditory cues and their relative impact on body ownership by, for instance, manipulating visuomotor and visuoauditory feedback independently during a motor task. A recent Hierarchical Sensory Predictive Control (HSPC) theory proposes a cascade of purely sensory predictions which mirror the causal sequence of the perceptual events preceding a sensory event (Maffei et al., 2017). In the context of anticipatory control, this control architecture acquires internal models of the environment and the body through a hierarchy of sensory predictions from visual (distal) to proprioceptive and vestibular modalities (proximal). If body ownership and motor control share the same forward models, which comprise both distal, proximodistal, and proximal signals, ownership might be realized through a similar cascade of sensory predictions. In our case, however, which includes a goal-oriented task and voluntary control, the internal models might be acquired from the proprioceptive and vestibular modalities (proximal) to visual (distal), a hypothesis yet to be investigated. In such case, one could expect differences in reaction times between the congruent and the incongruent conditions due to increased sensory prediction errors. Interestingly, our results yielded no differences in the reaction times between the groups. We believe that this result might depend on the congruency of proximodistal signals. Specifically, the visual feedback of the movement always matched the proprioceptive cues. It is possible that for motor control the prediction errors from the proximodistal modalities are more relevant (they are weighted higher) than those from purely distal. We suggest that future studies should systematically investigate the contribution of different cues to performance, possibly within the framework of HSPC (Maffei et al., 2017). Second, if body ownership depends on the consistency of internal models, and therefore on the accuracy of sensory predictions, could task-irrelevant signals manipulate it? While playing Air Hockey, the brain does not only integrate action-driven sensory signals but also simultaneously processes purely external action-independent information which derives from the environment. This information might well include corrective information and, therefore, be relevant to the task (i.e., the wind which affects the trajectory of the puck) or not (i.e., time of the day) (Shadmehr et al., 1994). Changing the rules of the environment and investigating the experience of ownership and performance when action-independent (task-irrelevant) sensory expectations are violated would shed light on the nature of sensory signals relevant for the processing of self as well as their underlying mechanisms (i.e., generative and forward models) (Friston, 2012; Seth, 2013; Apps and Tsakiris, 2014).

What is the role of purely distal action-driven cues in goal-oriented behavior? Our results demonstrate that performance, as measured through the overall scores (Figure 2A) and directional errors (Figures 2B, C), was significantly hampered in the incongruent compared to the congruent condition. Importantly, these results did not depend on differences in reaction times (Figure 2E) suggesting no influence of possible attentional biases (i.e., distractions) in either of the groups. On the one hand, this outcome might be interpreted within the framework of computational motor control. The reported differences in performance between the two conditions could have been influenced by the discrepancies between the efference copies of distal events and the actual action outcomes. Indeed, results from motor control studies support the notion that learning (progressive reduction of error) depends on both proximal and distal sensory prediction errors that allow for adjustments and anticipation of possible perturbations deriving from the body and environment (Jordan and Rumelhart, 1992; Mazzoni and Krakauer, 2006; Tseng et al., 2007; Krakauer, 2009; Maffei et al., 2017; Morehead et al., 2017). As a result, during action execution, inputs from all the sensory modalities are transformed into error signals updating the forward model and, consequently, future behavior (Wolpert and Kawato, 1998; Kawato, 1999; Shadmehr et al., 2010; Wolpert et al., 2011). In our experiment, the directionality of the error indicated by the spatial distribution of the sound, the speed of the puck indicated by the temporal characteristics of the sound, as well as the knowledge of results all constituted error signals which could supervise corrective motor commands. Crucially, while the spatial and temporal dimensions provided information about the action parameters on a continuous range, the semantic dimension constituted a binary reinforcement signal informing about a failure or a success. As such, the chosen audiovisual cues in the incongruent condition might have influenced performance, which, in turn, affected body ownership. In fact, clinical studies provide evidence that patients suffering from hemiparesis, whose motor function is reduced due to stroke, progressively stop using the paretic limb: the so-called learned non-use phenomenon (Taub et al., 2006). In this, and other neurological cases, a prolonged lack of use (low performance) often causes disturbances in the sense of ownership and agency (Gallagher, 2006) supporting a hypothesis that there might be a causal effect between performance and body ownership. The present design which includes three types of sensory manipulations pseudorandomly distributed within each block does not allow us to disambiguate between the specific contribution of each of the manipulations. A systematic study on the influence of individual sensory signals, including the three manipulations, would help to better understand the mechanisms accounting for low-performance scores in the incongruent condition. An alternative interpretation of our results is related to the experimental and theoretical framework of body ownership. Several studies propose that body ownership is coupled to the motor system such that it updates the sensory representation of the body and provides inputs to the forward model. The forward model, in turn, generates and updates predictions relative to both the body and the environment during voluntary actions (Kilteni and Ehrsson, 2017), reinforcing the history of sensorimotor contingencies. In particular, we find evidence that body ownership is involved in generating body-specific predictions about the sensory consequences of voluntary actions thus determining somatosensory attenuation (Kilteni and Ehrsson, 2017). This finding is consistent with another study which employed a standard RHI in virtual reality and demonstrated that ownership is correlated with motor performance during a perceptual decision-making task (Grechuta et al., 2017). Contrary to the previous discussion, in this case, ownership would have a modulatory effect on performance.

At the current stage, we cannot disambiguate between the two alternative hypotheses and determine whether the integration of purely distal cues influences ownership and performance in parallel or independently and what is the directionality. We demonstrate, however, that both depend on the congruency of action-driven and task-relevant purely distal signals, which supports the notion that both rely on the consistency of forward models driving goal-oriented action (Seth, 2013; Apps and Tsakiris, 2014). We expect that this outcome will allow for the advancement of our understanding of the mechanisms underlying body ownership. To improve the experimental quality of the present study and further support our findings, future studies shall consider a bigger sample size as well as an alternative objective measure of ownership (i.e., body temperature) which would allow for conducting a within-group experiment without biasing the physiological signals (Moseley et al., 2008). Finally, the reported finding might find applications in fields such as motor training simulators and rehabilitation. For instance, virtual reality-based treatments of post-stroke motor disorders (Cameirão et al., 2010; Grechuta et al., 2014; Mihelj et al., 2014; Ballester et al., 2015) might benefit from a design of reliable and spatiotemporally congruent environments which may positively impact the ownership of the virtual body as well as performance possibly impacting recovery. Further clinical studies should evaluate the same principle in rehabilitation protocols for ownership disturbances following acquired brain lesions including neglect (Coslett, 1998), anosognosia for hemiplegia (Pia et al., 2004) or somatoparaphrenia (Fotopoulou et al., 2011).

## Ethics Statement

The protocol was approved by the ethical committee of the University of Pompeu Fabra (Barcelona, Spain). All subjects provided their written informed consent.

## Author Contributions

KG, LU, and BR designed the protocol, KG and LU conceived the experiment and LU conducted the experiments, KG and LU analyzed the results, KG, LU, and PV wrote the manuscript. PV initiated and supervised the research. All authors reviewed and approved the manuscript.

### Conflict of Interest Statement

The authors declare that the research was conducted in the absence of any commercial or financial relationships that could be construed as a potential conflict of interest.
